# Feasibility and safety of ultrasound-guided percutaneous transhepatic measurement of portal venous pressure

**DOI:** 10.1371/journal.pone.0305725

**Published:** 2024-07-19

**Authors:** Yanshan Hu, Shaobo Duan, Ye Zhang, Liuwei Hao, Shuaiyang Wang, Fei Xue, Kewei Zhang, Yadong Zhu, Lianzhong Zhang

**Affiliations:** 1 Zhengzhou University People’s Hospital, Zhengzhou, China; 2 Henan Engineering Technology Center of Ultrasonic Molecular Imaging and Nanotechnology, Zhengzhou, Henan Province, China; 3 Department of Health Management, Henan Provincial People’s Hospital, Zhengzhou, Henan Province, China; 4 Department of Hepatobiliary Surgery, Henan Provincial People’s Hospital, Zhengzhou, Henan Province, China; 5 Department of Vascular Surgery, Henan Provincial People’s Hospital, Zhengzhou, Henan Province, China; 6 Department of Ultrasound, Henan Provincial People’s Hospital, Zhengzhou, Henan Province, China; Coventry University, UNITED KINGDOM

## Abstract

**Background and objective:**

The measurement of portal venous pressure (PVP) has been extensively studied, primarily through indirect methods. However, the potential of ultrasound-guided percutaneous transhepatic PVP measurement as a direct method has been largely unexplored. This study aimed to investigate the accuracy, safety, and feasibility of this approach.

**Methods:**

*In vitro*, the experiment aimed to select a needle that could accurately transmit pressure, had a small inner diameter and was suitable for liver puncture, and performed on 20 healthy New Zealand white rabbits. An ultrasound-guided percutaneous transhepatic portal vein puncture was undertaken to measure PVP. Additionally, free hepatic venous pressure (FHVP) and wedged hepatic venous pressure (WHVP) were measured under digital subtraction angiography (DSA). The correlation between the two methods was assessed. Enroll study participants from October 18, 2023 to November 11, 2023 with written informed consent. Five patients were measured the PVP under ultrasound guidance before surgery to determine the feasibility of this measurement method.

**Results:**

There was no significant difference in the results obtained using 9 different types of needles (*P* > 0.05). This demonstrated a great repeatability (*P* < 0.05). The 22G chiba needle with small inner diameter, allowing for accurate pressure transmission and suitable for liver puncture, was utilized for percutaneous transhepatic PVP measurement. There were positive correlations between PVP and HVPG (*r* = 0.881), PVP and WHVP (*r* = 0.709), HVPG and WHVP (*r* = 0.729), IVCP and FHVP (*r* = 0.572). The PVP was accurately and safely measured in 5 patients with segmental hepatectomy. No complications could be identified during postoperative ultrasound.

**Conclusion:**

Percutaneous transhepatic portal venous puncture under ultrasound guidance is accurate, safe and feasible to measure portal venous pressure.

**Clinical trial registration number:**

This study has been registered in the Chinese Clinical Trial Registry with registration number ChiCTR2300076751.

## 1. Introduction

Portal hypertension (PHT) is a medical condition marked by an abnormal increase in pressure within the portal vein, which can result in various complications, such as an enlarged spleen, the development of varicose veins in the esophagus and stomach, vomiting blood, accumulation of fluid in the abdomen, and impaired brain function. As per statistical data, liver cirrhosis was responsible for causing approximately 13.329 million fatalities globally during the year 2017. A significant proportion of individuals diagnosed with liver cirrhosis experienced the coexistence of esophageal and gastric varices, with a further 50% to 60% of these cases being complicated by severe hemorrhages, resulting in a high mortality rate [1–3]. Currently, the hepatic venous pressure gradient (HVPG = WHVP—FHVP) serves as the parameter for evaluating portal venous pressure (PVP), with a standard range of 3–5 mmHg.

Accurate measurement techniques are crucial for diagnosing PHT. Myers and Taylor were the first to propose using wedged hepatic venous pressure (WHVP) as an indirect indicator of PVP [4]. The normal range for PVP is 13–24 cmH_2_O(1 cmH_2_O = 0.736 mmHg) [5–7]. This approach has been instrumental in revealing the pathophysiological mechanism [8], while it also has well-known limitations [9–12]. Splenic pulp manometry or transumbilical portography techniques have become irrelevant with the advent of HVPG. Additionally, the limited availability of HVPG measurement in medical centers, along with its demanding requirements for skilled operators and risk of radiation exposure, has hindered its widespread adoption in clinical practice [13–16].

As technology advances and research deepens, the investigation of portal vein puncture for blood sampling and pressure measurement, studies [17–20] have not revealed compelling evidence of complications, such as bleeding, embolism, or arteriovenous fistula caused by the use of a 16-22G needle. PVP measurement tools are shifting from the initial polyethylene straight catheter to the more advanced balloon catheter [21, 22] and the chiba needle. Similarly, the methods used for puncture positioning and guidance have evolved from relying on X-ray to the assistance of DSA technology. Additionally, ultrasound technology has found its application in puncture biopsy, radiofrequency ablation of liver tumors, and real-time needle passage hemostasis, contributing to the exploration of PVP measurement. The use of hemostatic materials (e.g., collagen, spring coil, N-butyl cyanoacrylate or gelatin sponge, etc.) combined with ultrasound significantly lowers the potential risk of bleeding [23, 24], and provides a method for directly measuring PVP. This investigation was carried out to examine the safety, viability, and precision of ultrasound-guided transhepatic PVP measurement.

## 2. Instruments and materials

### 2.1 Experimental instruments

The following instruments were utilized: peristaltic pump (LONGER L100-1S-1, Lange Constant Flow Pump Co., Ltd., China), monitors (PHILIPS IntelliVue MP20, Netherlands; mindray BeneVision N22, China), ultrasound machine (mindray Resona 8S), and digital subtraction angiography (DSA) machines (GE Healthcare, USA).

### 2.2 Experimental subjects

#### 2.2.1 Animal experiment

20 healthy male New Zealand white rabbits.

#### 2.2.2 *In vivo* experiment

Five patients who would receive segmental hepatectomy by laparotomy. Before surgery, methylene blue was injected into the hepatic portal vein to label the hepatic segments.

## 3. Methods

### 3.1 Puncture needle selection through an *in vitro* model

An experimental model was created to mimic the anatomical and hemodynamic characteristics of human blood vessels for *in vitro* studies. The blood pumping process was simulated using a peristaltic pump, while two fluid media (0.9% sodium chloride injection and hydroxyethyl starch injection) were used to mimic blood flow. The model blood vessel was divided into inner and outer 1/2 regions, and the peristaltic pump speed was adjusted to 20, 25, and 30 rpm. To measure fluid dynamics, nine different types of puncture needles with various inner diameters and opening holes were used to collect data in both the inner and outer regions of the model. A pressure transducer connected to a monitor was used for data recording. The measurement data obtained from the seven different needle types were statistically analyzed in comparison to the BD-24G/20G (0.7 mm/1.1 mm) needle. Based on the statistical analysis of intraclass correlation coefficient (*ICC*), a needle with smaller diameter and suitable for liver puncture was selected for follow-up study.

### 3.2 Animal experiment

**Fig 1:**
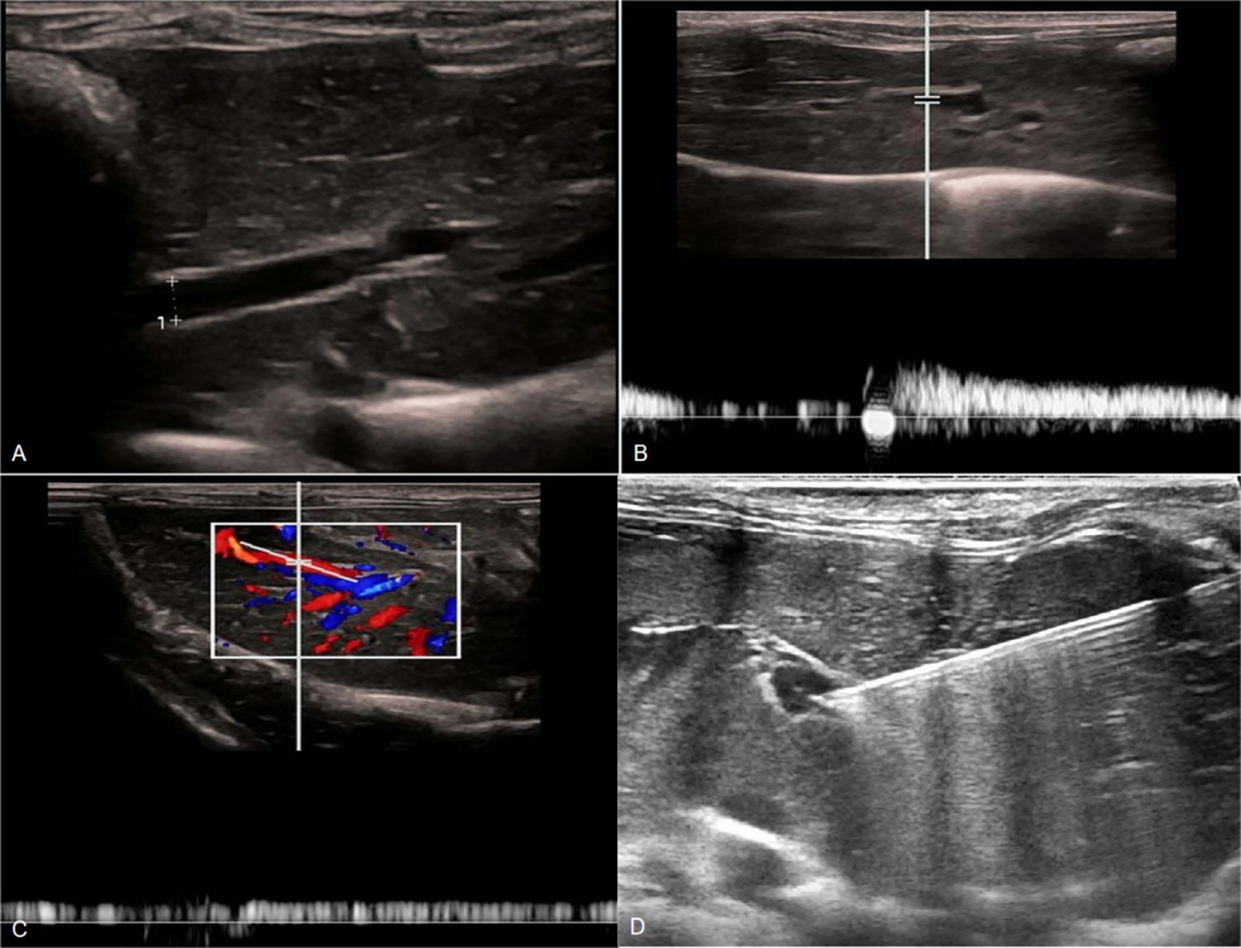
Measurement of inner diameter, flow rate, and pressure of portal vein under ultrasound guidance. A: Measurement of inner diameter of portal vein; B, C: Measurement of flow rate of portal vein; D: Measurement of PVP by puncture.

**Fig 2:**
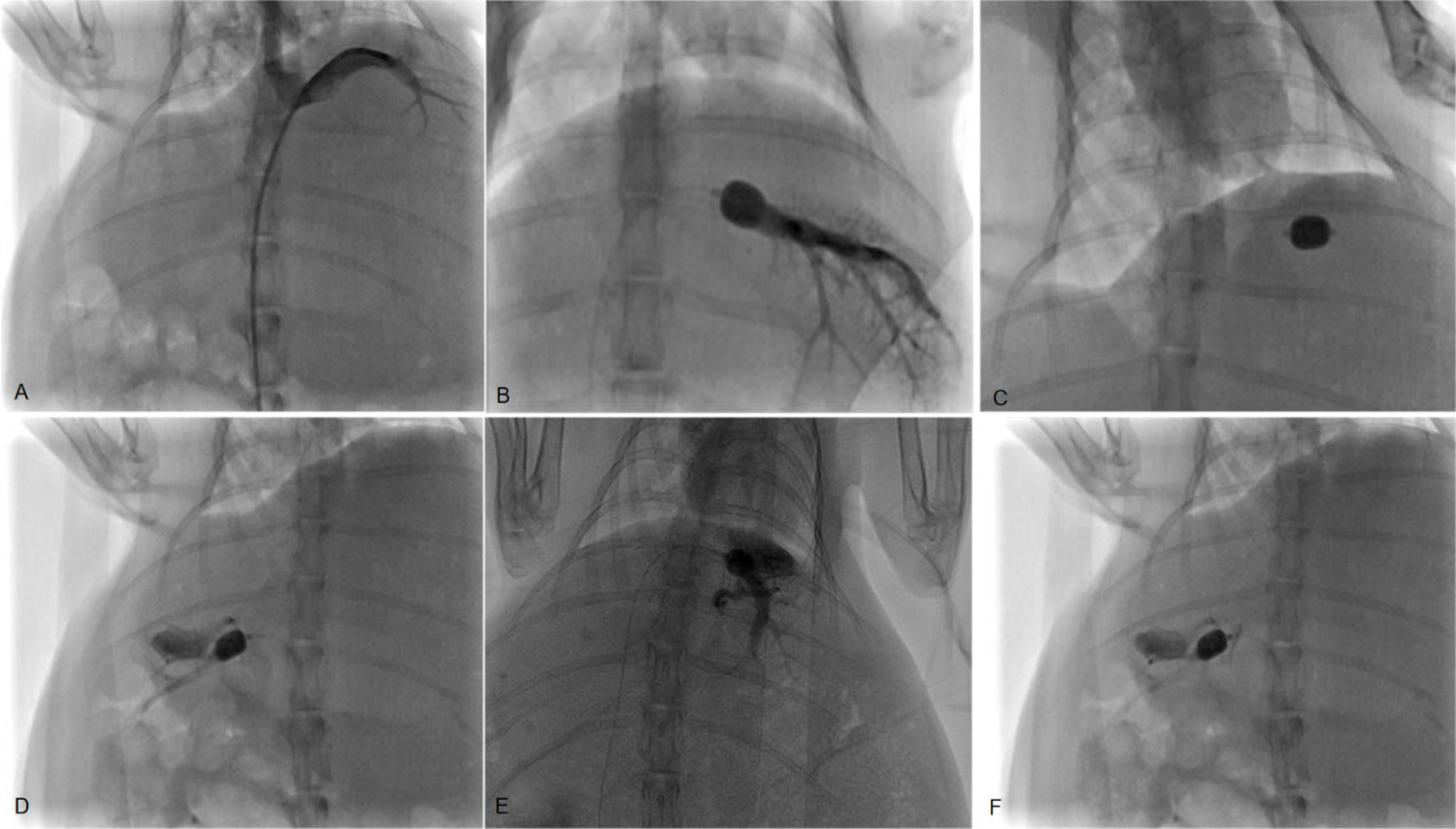
HVPG measurement under DSA. A: The angiographic catheter was successfully introduced to the branches of the hepatic vein, and angiography was performed to exclude shunts, leakage, and collateral circulation, etc. B: The balloon catheter was wedged into the hepatic vein, and the balloon was injected with the contrast agent. The contrast agent was injected to the hepatic vein through the tip of the catheter. The balloon was dilated to check for any contrast agent leakage or reflux. C: 2 mL of contrast agent was slowly injected via the balloon catheter. Using the balloon dilated, after withdrawal until blood was observable, normal saline was injected to drain the blood and contrast agent in the balloon catheter, and the balloon catheter was connected with the pressure transducer for pressure measurement. D, E, and F: Angiography confirmed the presence of obvious veno-venous collateral shunts.

A total of twenty male New Zealand white rabbits, which were sourced from the Animal Experiment Center and had an average weight of 2.485 ± 0.296 kg, were utilized. This experiment was carried out in strict accordance with the recommendations in the Guide for the Care and Use of Laboratory Animals of the National Institutes of Health. The experimental protocol was approved by the Animal Ethics Committee of the Animal Experiment Center at Zhengzhou University under the code ZZU-LAC20220428(01). To initiate the experiment, rabbits first underwent general anesthesia using sevoflurane at concentrations of 1.2% to 3%. Subsequently, the ultrasound was employed to measure the inner diameters and flow rates of the main portal vein as well as its left and right branches (Fig 1). Using guidance from the ultrasound, a percutaneous transhepatic puncture was performed using the needle selected in *vitro* model study to determine the PVP. WHVP and FHVP were measured by femoral vein under DSA (Fig 2). HVPG was calculated using the formula HVPG = WHVP–FHVP (S1 Appendix). After the procedure, ultrasonography was performed on the liver to check for any complications, such as bleeding. All procedures are performed under sevoflurane anesthesia and every effort is made to reduce pain.

### 3.3 *In vivo* experiment

**Fig 3:**
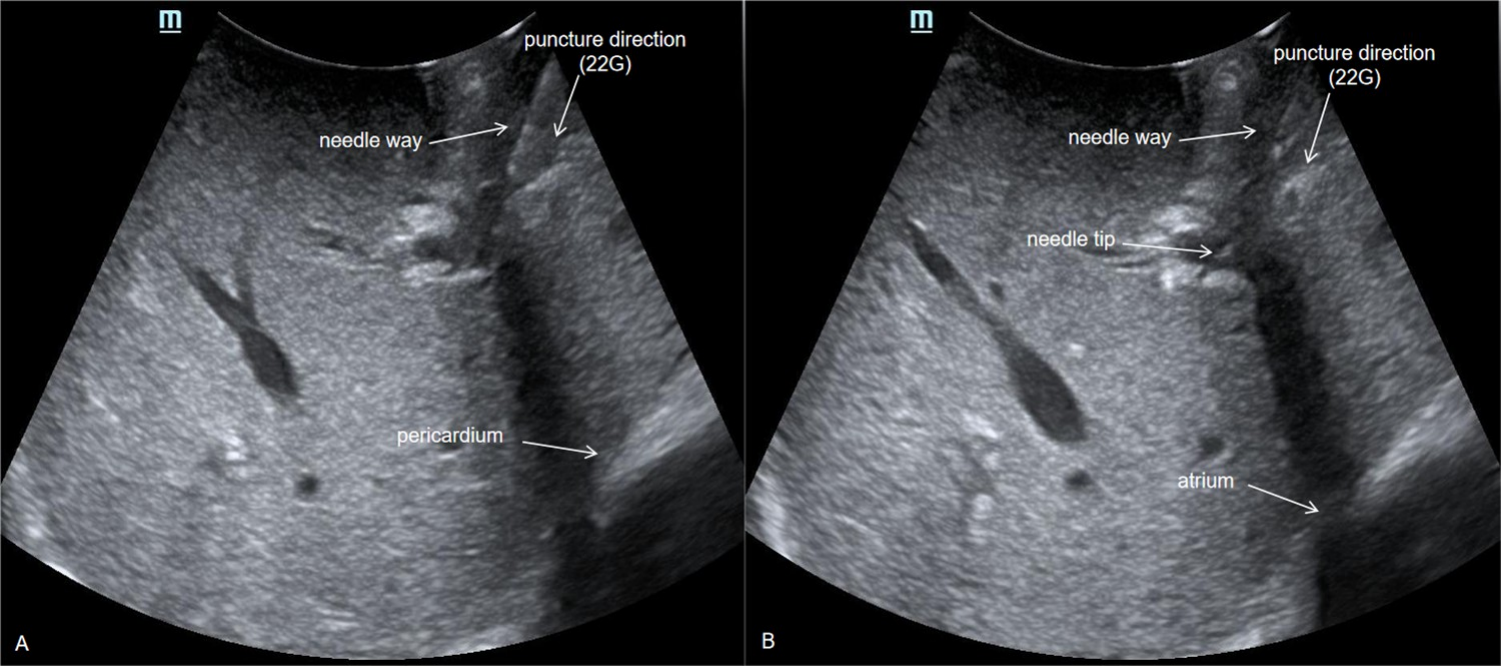
Intraoperative ultrasound-guided PVP measurement image.

Enroll study participants from October 18, 2023 to November 11, 2023 with written informed consent. The subjects for this study consisted of five patients who were scheduled to undergo segmental hepatectomy by laparotomy. The protocol was approved by the Medical Ethics Committee of Henan Provincial People’s Hospital of Zhengzhou University, with the ethics number of 112 registered in the year 2022. After patients were administered general anesthesia, and ultrasonography was utilized to measure the inner diameters and flow rates of either the main portal vein or its branches. Prior to the surgical procedure, methylene blue was injected into the hepatic portal vein using the needle selected in *vitro* model study to mark the hepatic segments. To prevent any bubbles, approximately 2 mL of normal saline was flushed into the needle passage following the methylene blue injection. The pressure measurement is performed before the needle withdrawal. Holographical data, such as PVP, were recorded (Fig 3).

## 4. Statistical analysis

The statistical analysis was conducted using SPSS software, specifically version 25.0 released by IBM headquartered in USA. For data that followed a normal distribution, the metric of mean ± standard deviation (SD) was employed. For data that did not conform to a normal distribution, the median and interquartile range (IQR) would be utilized. The *ICC* measure was employed to assess the consistency of pressure measurements and the reliability of repeated measurements. It was attempted to draw a scatter plot to display the correlation between the two methods of measurement, and Pearson’s correlation coefficient (*r*) was calculated. A significance level of *P* < 0.05 was deemed statistically significant.

## 5. Results

### 5.1 *In vitro* model

It was revealed found that the outcomes obtained using 9 different types of needles in the same position along or against the fluid direction did not show any significant differences (*P* > 0.05).

**Fig 4:**
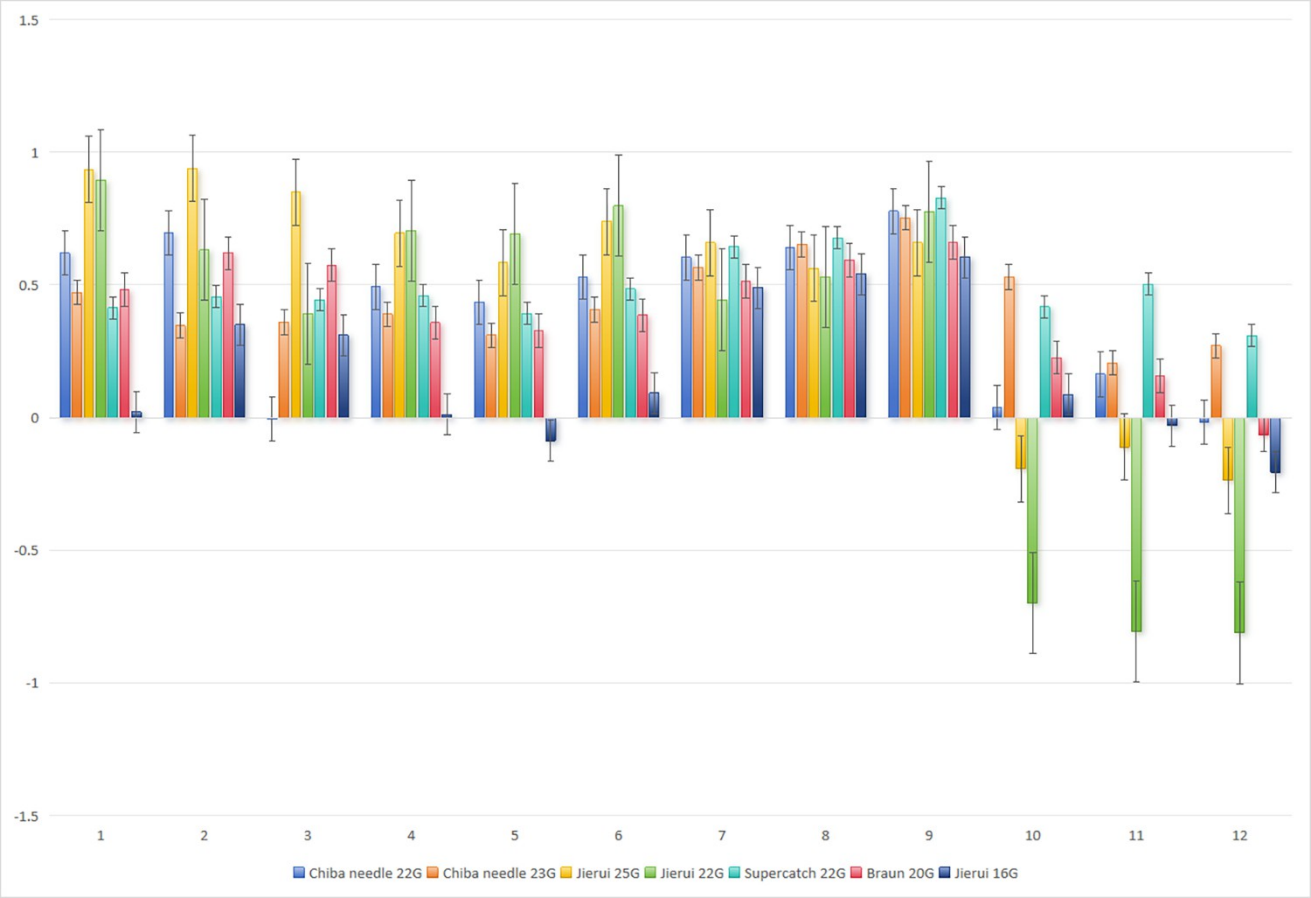
Take BD-24G as the reference standard. Based on the statistical analysis of intraclass correlation coefficient. Validation and selection of punture needle.

**Fig 5:**
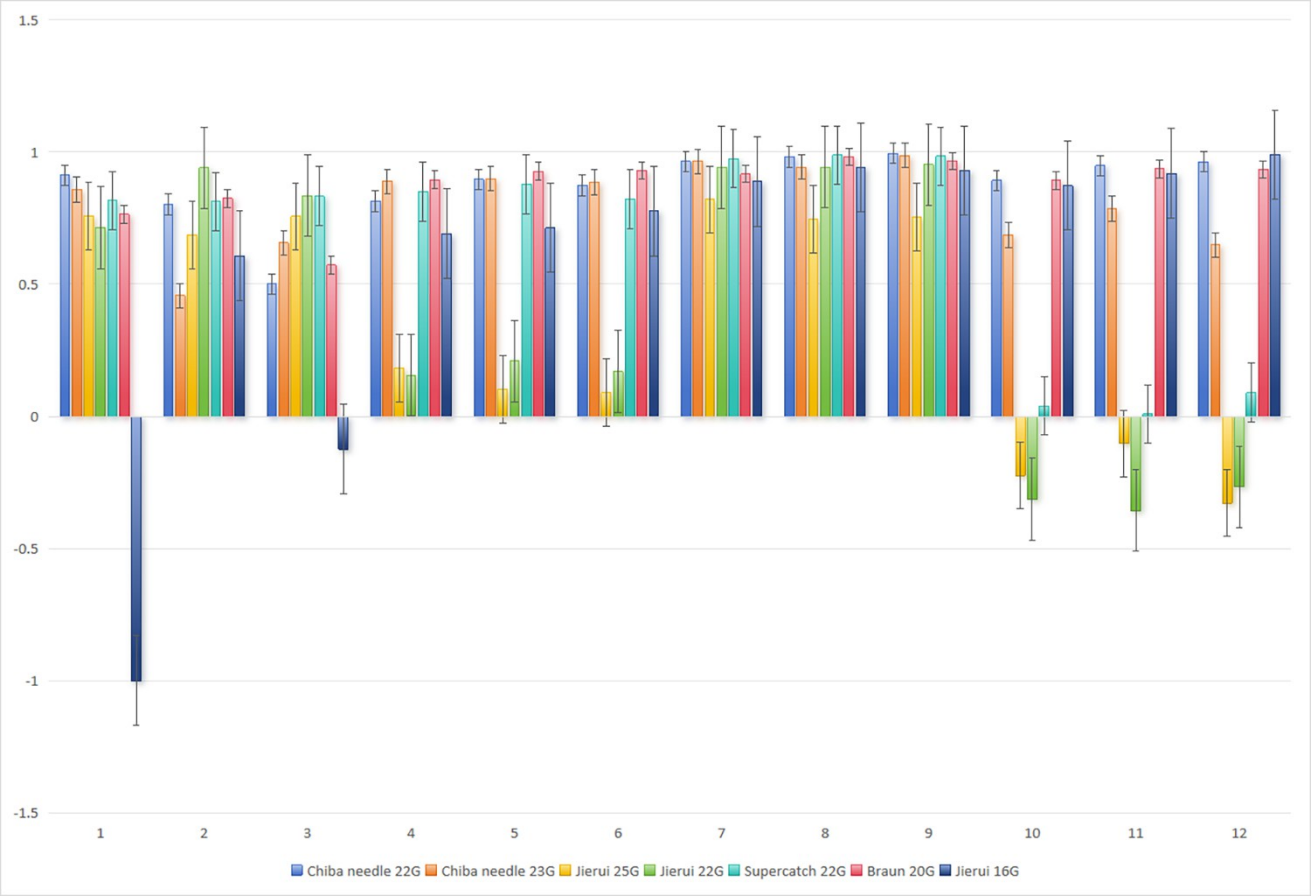
Take BD-20G as the reference standard. Based on the statistical analysis of intraclass correlation coefficient. Validation and selection of puncture needle.

**Fig 6:**
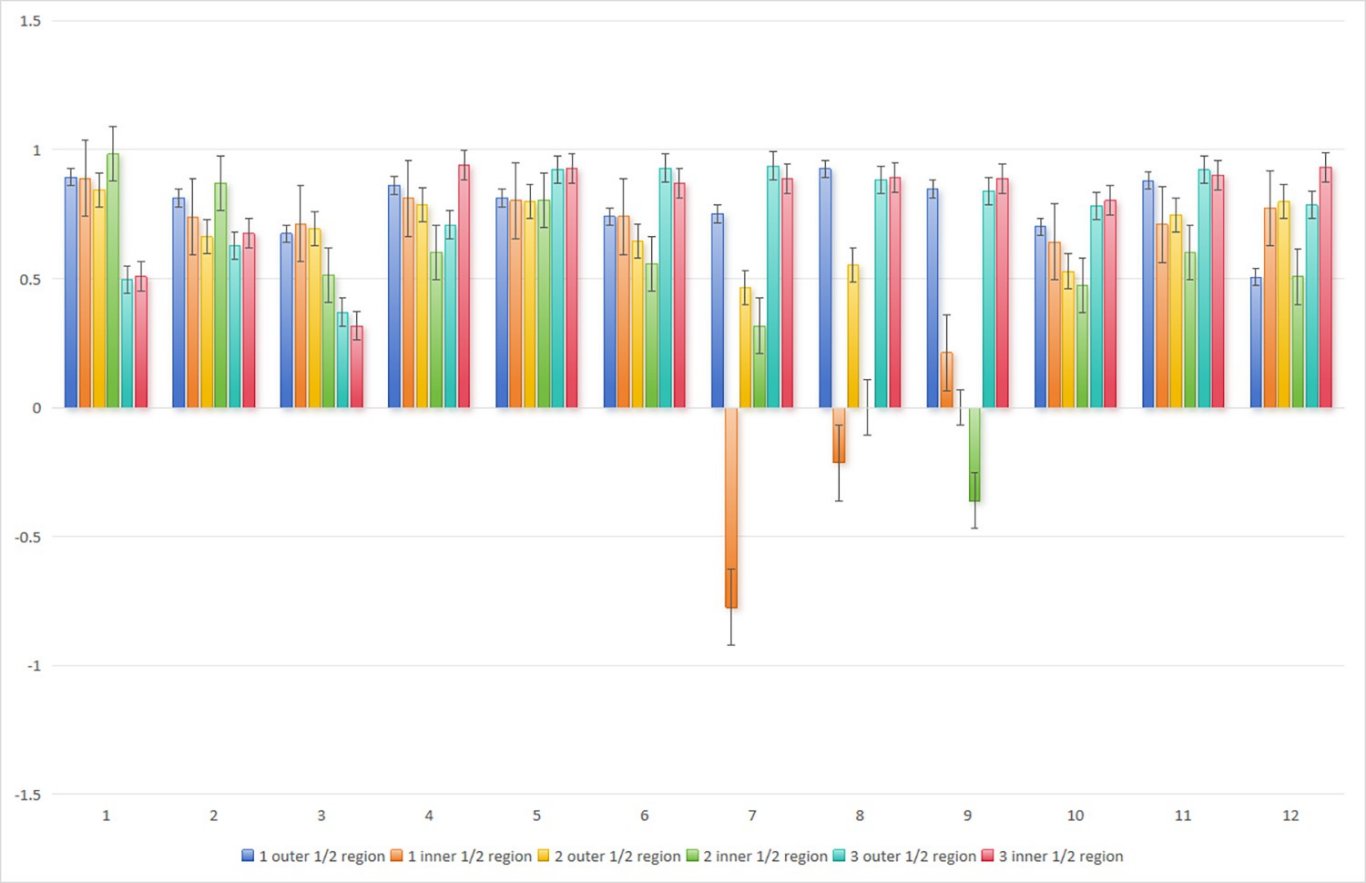
Repeated measurement reliability analysis. 1 is the proximal end; 2 is the center of proximal and distal end; 3 is the distal end. Notes: 1. single first branch (normal saline, 20 rpm); 2. single first branch (normal saline, 25 rpm); 3. single first branch (normal saline, 30 rpm); 4. single first branch (Voluven, 20 rpm); 5. single first branch (Voluven, 25 rpm); 6. single first branch (Voluven, 30 rpm); 7. two first branches in parallel (Voluven, 20 rpm); 8. two first branches in parallel (Voluven, 25 rpm); 9. two first branches in parallel (Voluven, 30 rpm); 10. two first branches in series (Voluven, 20 rpm); 11. two first branches in series (Voluven, 25 rpm); 12. two first branches in series (Voluven, 30 rpm).

For *in vitro* model data, the *ICC* of the correlation analysis was calculated with the BD needle (BD-24G (0.7 mm) and BD-20G (1.1 mm)) as the standard: In Fig 4, with BD-24G as the standard, the *ICC* was minimally -0.003 and maximally 0.777 for the chiba needle 22G, and minimally 0.206 and maximally 0.753 for the chiba needle 23G. In Fig 5, with BD-20G as the standard, the *ICC* was minimally 0.50, with other values above 0.8, and maximally 0.981 for the chiba needle 22G, and minimally 0.457, with other values above 0.6, and maximally 0.987 for the chiba needle 23G, demonstrating a remarkable correlation. Fig 6 shows the *ICC* (*r*) of the repeated measurement reliability analysis, in which the other 9 types of needles were utilized to measure the pressure in the inner and outer 1/2 regions along or against the fluid direction, respectively. Three pairs (3/72, 4.2%) of model data showed *P* > 0.05, and the other 69 pairs (69/72, 95.8%) of model data exhibited *P* < 0.05, indicating acceptable repeatability. Based on the above mentioned statistical results, we validated and selected the 22G chiba needle which is capable of accurate pressure transmission and suitable for liver puncture.

### 5.2 Animal experiment

A total of 17 New Zealand white rabbits were used in the experiment, resulting in the acquisition of 17 sets of data. The average PVP was (8.33 ± 1.62) mmHg. Measurements taken under DSA included WHVP (9.68 ± 2.11) mmHg, FHVP (6.48 ± 1.56) mmHg, HVPG (3.21 ± 2.12) mmHg, and IVCP (5.94 ± 1.58) mmHg.

**Fig 7:**
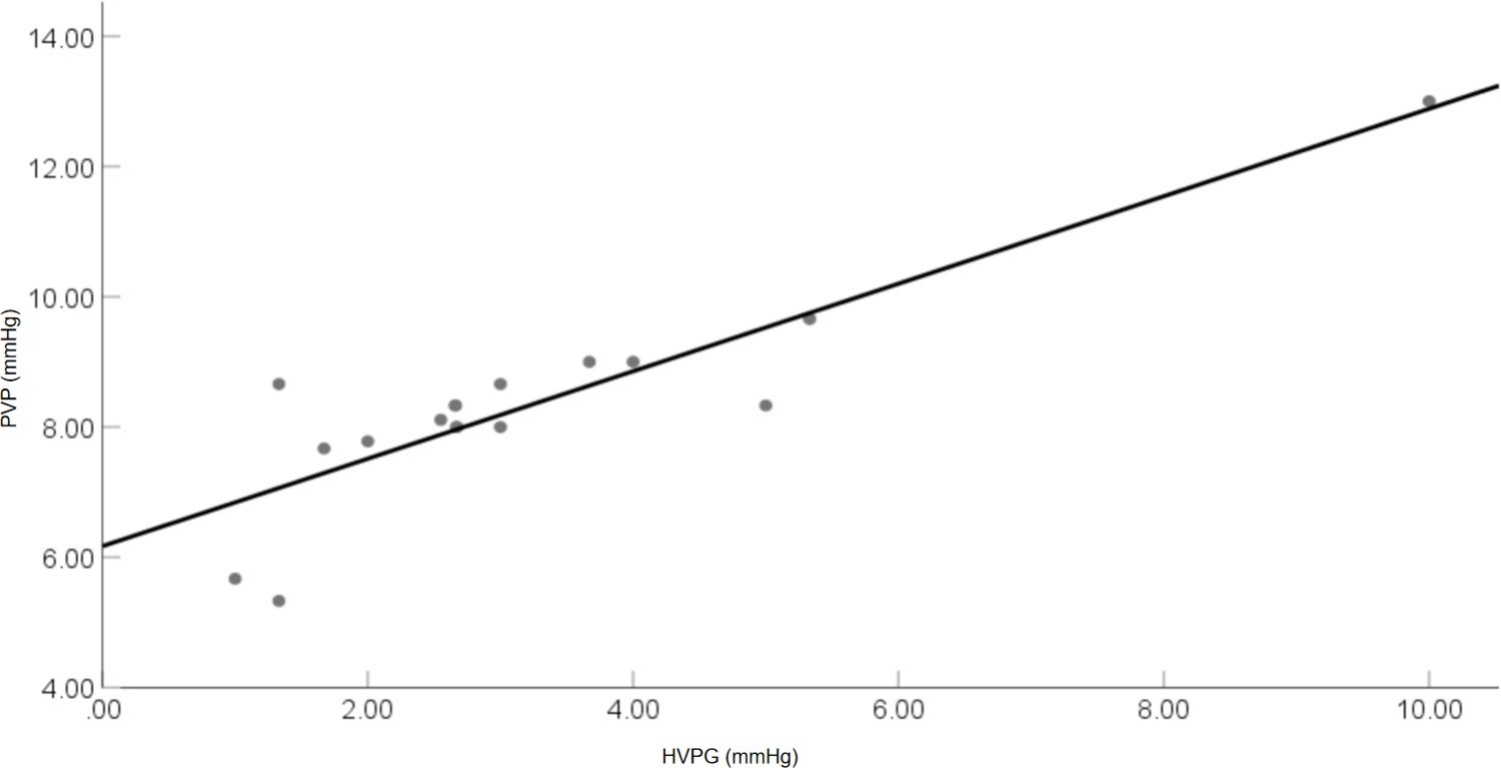
Correlation between portal venous pressure and hepatic venous pressure gradient (1 mmHg = 0.133 kPa).

5.2.1 There was a significantly positive correlation between PVP and HVPG, with a correlation coefficient of 0.881 (*P* < 0.01) (Fig 7). The linear regression equation that best fit the data was PVP = 6.171 + 0.672 × HVPG, with an R-squared value of 0.777. The slope of the regression line was 0.672. In Fig 7, the relationship between PVP and HVPG is depicted. The average HVPG and PVP are respectively (3.21 ± 2.12) and (8.33 ± 1.62) mmHg, indicating that PVP was, on average, higher than HVPG by 5.12 mmHg.

**Fig 8:**
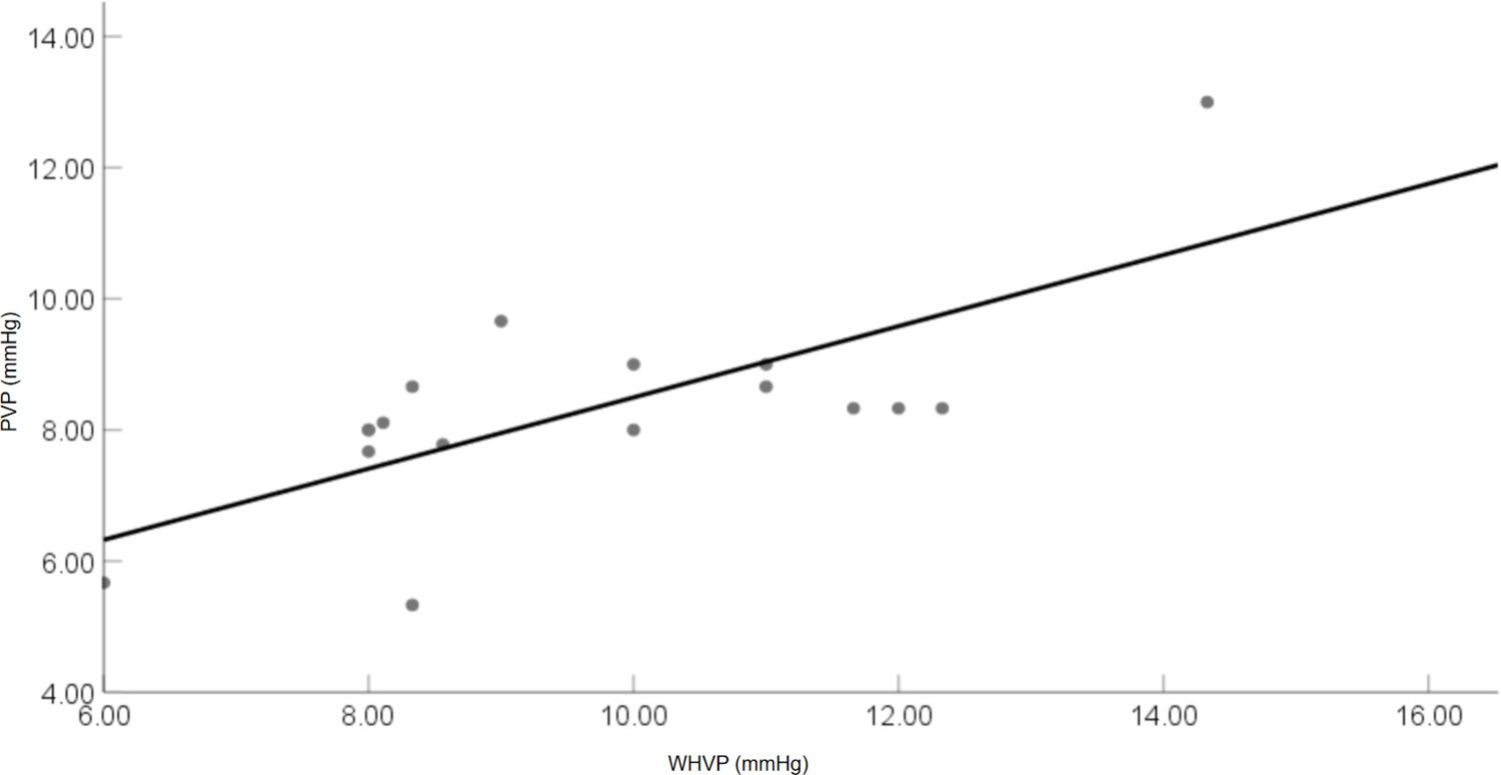
Correlation between portal venous pressure and wedged hepatic venous pressure (1 mmHg = 0.133 kPa).

5.2.2 There was a significantly positive correlation between PVP and WHVP, with a correlation coefficient (*r*) of 0.709 and a P-value of less than 0.01 (Fig 8). The linear equation that best fit the data was PVP = 3.066 + 0.543 × WHVP, with an R-squared value of 0.503, indicating that 50.3% of the variability in PVP could be explained by WHVP. The slope of the regression line was 0.543. There were positive correlations between HVPG and WHVP (*r* = 0.729, *P* < 0.01), IVCP and FHVP (*r* = 0.572, *P* < 0.01). The average WHVP was 1.35 mmHg higher than the mean PVP.

### 5.3 Clinical trial

The subjects selected for the study were 5 patients who were scheduled to undergo segmental hepatectomy through laparotomy. Once under general anesthesia, the internal diameter was measured using ultrasound at various locations. The results were as follows: (13.64 ± 0.096), (5.93 ± 0.221), (4.20 ± 0.156), (4.12 ± 0.130), and (5.92 ± 0.123) mm. Additionally, the PVP was measured and yielded the following values: (8.297 ± 0.65), (6.590 ± 1.11), (14.761 ± 0.85), (5.733 ± 0.94), and (15.00 ± 1.09) mmHg for each patient respectively. PVP in patients with portal hypertension was consistent with the clinical manifestations of esophageal and gastric varices and collateral circulation. Ultrasonography was performed in all patients after operation, and there were no complications such as obvious bleeding.

## 6. Discussion

HVPG, which is an indirect indicator of PVP, effectively assesses the hepatic sinusoidal pressure [25, 26]. This measurement holds significant value in the identification of the causes of PHT, as well as predicting the prognosis of chronic liver disease, gastrointestinal bleeding, and the effectiveness and outcome of drug treatments [13, 14, 27, 28]. Despite being an invasive evaluation technique, HVPG measurement has proven to be invaluable in clinical practice over the last few decades. However, its clinical application is hindered by its limitations and the need for skilled operators in medical centers [29–33]. PHT is categorized as pre-sinus, sinus, or post-sinus based on its underlying causes [34]. In cases of non-alcoholic liver cirrhosis, WHVP tends to underestimate PVP, highlighting the importance of directly measuring PVP [9]. The precision of measuring pressure directly is contingent upon several factors, including the careful selection of the position at which pressure is measured, the use of a needle that can effectively transmit pressure accurately, and the availability of real-time guiding and monitoring equipment like ultrasound.

We consistently affirmed the accuracy of pressure transmission through the needles using an artificial model, and the results indicated that there was no significant difference in the pressure recorded at the same position in either the same or opposite direction of the fluid flow (*P* > 0.05). This finding demonstrated a high level of consistency (*P* < 0.05) and great repeatability in the measurements obtained. After assessing the outcomes, the 22G chiba needle was selected as the needle for pressure measurement in both the animal experiment and the clinical trial. Due to the current conditions, the other needles are not the best needles for liver puncture in terms of material, hardness and length, because the angle needs to be adjusted in time to avoid important blood vessels and bile ducts during liver puncture, the smaller inner diameter of the needle and the insufficient toughness of the material, the needle will be bent and deformed during the puncture process, such as BD-24G, resulting in the failure to complete the operation smoothly. The increase in the inner diameter of the needle can lead to a greatly increased risk of bleeding, such as BD-20G. The failure of three other White Rabbits in the experiment may have something to do with it. Therefore, needles other than 22G chiba needle are not used in Animal Experiment and Clinical Trial for the time being.

During the animal experiment phase, while HVPG and WHVP indirectly indicate PVP [35], direct measurement of PVP offers a more direct assessment of PVP. Direct measurement remains to be effective and promising, particularly for pre-hepatic portal hypertension, pre-sinus portal hypertension (e.g., schistosomal or idiopathic portal hypertension), and cases involving pre-sinus components [36, 37]. The results indicated a strongly positive correlation between PVP and HVPG (*r* = 0.881, *P* < 0.01), suggesting that HVPG could be utilized to predict PVP in this species. Additionally, the two measurement methods showed high consistency. Among the methods employed for accurately measuring PVP, the direct measurement method could be utilized to evaluate PHT. PVP also displayed a positive correlation with WHVP (*r* = 0.709, *P* < 0.01), while IVCP exhibited a positive correlation with FHVP (*r* = 0.572, *P* < 0.01). Prior research demonstrated that in normal cats, the correlation coefficient between PVP and WHVP was *r* = 0.77 (*P* < 0.01) [38].

The reason for the mean WHVP being 1.35 mmHg higher than the mean PVP was not easily understandable. Previous research has consistently demonstrated that the mean WHVP in healthy dogs was consistently higher than the mean PVP [39]. Additionally, in some cases with liver cirrhosis, the WHVP has been found to be higher than the PVP, possibly due to factors, such as reverse hepatic blood flow, portal venous thrombosis or tumor thrombus, or abnormal venous shunting [9, 40].

There was no observation of blood flow in the needle passage when scattering the PVP during ultrasound-guided pressure measurement. Aside from abandoning some wedged branches with collateral circulation under DSA, no other phenomena were noted. In this study, the PVP was consistently lower than the WHVP by more than 3 mmHg in two animals, with a maximum difference of 4 mmHg. This discrepancy could potentially be attributed to the following reasons. Firstly, the direction of blood flow in the hepatic sinusoid might play a role. When the hepatic vein was wedged to block the flow, no reverse hepatic blood flow was generated within the normal hepatic sinusoid tissue. As a result, the static pressure created in this space did not diffuse through the microcirculation of various communicating and collateral branches within the hepatic sinusoid. Alternatively, when the blood flow was redirected to an adjacent hepatic sinusoid, it might cause a decrease or even complete cessation of flow within the hepatic sinusoid space. Secondly, if we do not take into account the possibility of free reflux into the portal vein system when the PVP would be exceeded, the increase in pressure at the tip of the balloon catheter could be due to an arterial factor [41].

Typically, FHVP is marginally higher than IVCP under normal circumstances. However, in this investigation, FHVP was either higher than or equal to IVCP in the majority of cases, and a positive correlation between the two was observed (*r* = 0.572, *P* < 0.01). Instances where FHVP was slightly lower than IVCP were carefully examined to exclude any measurement errors that could account for the disparity. Radiography findings indicated a close proximity between the hepatic vein and the right atrium, while compression of the abdominal intestine against the inferior vena cava in animals was identified as a significant contributing factor. If these abnormal outcomes were disregarded, the average FHVP would surpass IVCP by 1.24 mmHg, leading to a stronger correlation between IVCP and FHVP (*r* = 0.762, *P* < 0.01). These findings align with earlier studies in a theoretical sense [40, 42, 43]. In the experiment’s aftermath, it was revealed through ultrasonography that all animals successfully survived with no noticeable bleeding or complications. The use of direct measurement for PVP in medium-sized animal experiments proves to be effective, and employing the 22G chiba needle for pressure measurement enhances safety [44–47]. This study, conducted on New Zealand white rabbits, represents the first investigation into PVP and HVPG measurement in this particular animal species. We collected important data on normal New Zealand white rabbits, including the inner diameters and flow rates of the main portal vein, as well as the left and right branches. Additionally, we measured WHVP, FHVP, IVCP, and observed normal hepatic pathology. These findings can serve as a reference for future studies on portal hypertension in medium-sized animal models. Further research on PPV and HVPG in New Zealand white rabbits holds potential value in the future.

It has been documented that in typical situations, FHVP is generally slightly elevated by 0.5–1.0 mmHg compared to IVCP in humans [7, 34]. This discrepancy between PVP and IVCP indirectly indicates the pressure distinction between the portal vein and the inferior vena cava, known as the portal vein pressure gradient (PPG). While there may not be many studies linking HVPG and PPG [26], it is possible to measure PPG through endoscopic ultrasound-guided portal vein puncture, and ultrasound-guided transhepatic PVP measurement offers a more direct approach. We utilized a 22G chiba needle to perform ultrasound-guided PVP measurement in patients scheduled for segmental hepatectomy by laparotomy. Although we were unable to measure HVPG simultaneously under DSA due to ethical constraints, the result obtained through ultrasound-guided transhepatic portal vein puncture represents the accurate net pressure at the end of the hepatic sinus bed. This method is more convenient than using HVPG and WHVP and can assist in selecting appropriate intraoperative surgical techniques [48]. Therefore, measuring PVP directly can provide a more accurate reference point for diagnosing, distinguishing between conditions, determining severity, and assessing postoperative prognosis for PHT [49, 50]. Additionally, the advantages of direct PVP measurement, such as not requiring contrast agents or exposing patients to radiation, easy operation, and lower economic cost, are beneficial for patients and demonstrate its clinical value.

Our conclusion is that the accurate measurement of PHT depends on its etiology. To ensure accuracy, it is necessary to combine the advantages of different measurement methods to avoid misleading results from a single method. This study has some limitations, such as a small sample size and exclusion of patients with severe abdominal pressure disturbances (e.g., obesity, ascites, and splenomegaly) who did not undergo laparotomy. While laparotomy was used to exclude abdominal pressure interference in this study, PVP may still be affected by fluctuations in the abdominal aorta and respiratory movement of the diaphragm in patients with incompletely dissociated liver ligaments. Therefore, controlling or eliminating these factors remains crucial for accurate pressure measurement. Additionally, we performed ultrasonography on the liver after pressure measurement and did not observe any bleeding or complications.

## 7. Conclusions

There was no statistical difference in the pressure measurement results of 9 kinds of needles at the same position, and the consistency and repeatability were good. It is accurate and safe to employ 22G chiba needle with small inner diameter and suitable for liver puncture for ultrasound-guided percutaneous transhepatic portal vein puncture to measure pressure in animal experiments. And this method can be applied for clinical practice.

## Supporting information

S1 Appendix Operating procedures for hepatic venous pressure gradient measurement in New Zealand white rabbits. (DOCX)

## Abbreviation

PVP: Portal Venous Pressure
FHVP: Hepatic Venous Pressure
WHVP: Wedged Hepatic Venous Pressure
IVCP: Inferior Vena Cava Pressure
HVPG: Hepatic Venous Pressure Gradient
DSA: Digital Subtraction Angiography
PHT: Portal Hypertension
PPG: Portal Vein Pressure Gradient
IQR: InterQuartile Range
ICC: Intraclass Correlation Coefficient
