# Supplementary material for: Feasibility and safety of ultrasound-guided percutaneous transhepatic measurement of portal venous pressure
Source: PLoS One. 2024 Jul 19;19(7):e0305725. doi: 10.1371/journal.pone.0305725 (PMC11259298; doi:10.1371/journal.pone.0305725)

**Appendix: Operating Procedures for Hepatic Venous Pressure Gradient** **Measurement in New Zealand White Rabbits**

**1. Instrument setup and pressure measurement recording**

(1) Continuity and stability of pressure measurement should be ensured for standard testing. Therefore, we used a PHILIPS MP20 monitor to record the data.

(2) A venous pressure range of 0-50 mmHg was used during the measurement.

(3) The values were recorded after the pressure was persistently stable.

**2. Methods**

(1) Preparation before pressure measurement: The pressure transducer was connected to the monitor, and the sterile normal saline was used to evacuate air in the connecting pipe. The transducer and the connected tee joint were fixed at the atrial level, and the tee joint was rotated to connect to the air for zero calibration. Zero calibration should be repeated in case of any movement during measurement. The integrity of the Fogarty balloon catheter (model: 3F, 1.00 mm) was checked.

(2) Puncture process: The femoral vein of the white rabbit was selected. After routine disinfection, draping, and successful puncture, the catheter sheath was inserted. The angiographic catheter (an elbowed catheter or another multi-purpose catheter, as shown in Figure 2-A) was introduced into the hepatic vein through the inferior vena cava using a guide wire. The angiography confirmed that the vein was patent without stenosis or obvious veno-venous collateral shunts (Figure 2-B, D, E, and F). Thus, the hepatic vein could be selected for pressure measurement (otherwise, it would be replaced with another hepatic vein). The guide wire was introduced again, the catheter was withdrawn and the balloon catheter was introduced to the entrance of the hepatic vein by the guide wire. The guide wire was withdrawn. After withdrawal until blood was observable, normal saline was injected to drain the blood and contrast agent in the balloon catheter, and the balloon catheter was connected with the pressure transducer, during which, bubbles should be avoided.

(3) Measurement of free hepatic venous pressure (FHVP)

The balloon catheter was introduced to the hepatic vein under DSA and kept for at least 20 s at 2 cm to the inferior vena cava. The FHVP was read until the pressure value was stable.

(4) Measurement of wedged hepatic venous pressure (WHVP)

The contrast agent was injected to dilate the balloon to fully block the blood flow in the hepatic vein. After 30 s until the displayed pressure value was stable, the WHVP was read. Using the balloon dilated (as shown in Figure 2-C), 2 mL of contrast agent was slowly injected through the balloon catheter to confirm the absence of contrast agent reflux or veno-venous collateral shunts.

(5) FHVP and WHVP were measured repeatedly. The mean value of three readings was used.

(6) HVPG was calculated according to the formula (HVPG = WHVP – FHVP), and the mean value of two results was used.

(7) Inferior vena cava pressure (IVCP) was measured after the balloon catheter was retracted to the inferior vena cava at 2 cm below the entrance of the hepatic vein.

(8) Postoperative matters

1) After measurement, remove the catheter and sheath, timely perform pressure dressing with sterile gauze, and press the puncture point for 5-10 min.

2) Check for abdominal bleeding or hematocele, puncture point bleeding, or peripheral hematoma under ultrasound guidance.

3) Make the white rabbits awake and observe in time.

(9) Precautions

1) Aseptic operation should be strictly performed during pressure measurement to prevent blood-borne infections.

2) Air should be strictly avoided during puncture, zero calibration and device placement to prevent air embolism from affecting measurements.

3) The position of zero point should be checked before each pressure measurement to prevent position shifting.

4) The balloon catheter should be selected according to the inner diameter of the hepatic vein, and Fogarty balloon catheter (3F 1.00 mm) should be selected for the experimental group of New Zealand white rabbits.

5) Before each measurement, the balloon catheter should be filled with sterile normal saline to drain the air, blood and contrast agent, to avoid any potential impact on pressure measurement.

6) The appropriate anesthesia, instead of too light or deep anesthesia, should be maintained for the white rabbits. Insulation measures should be taken, to avoid body position change during the measurement.

7) If the catheter is blocked, it should be withdrawn until blood is returned, and rinsed with heparin saline, but not under excessive pressure. A new catheter should be replaced if necessary.

8) The maximum dose of contrast agent injected into the balloon catheter should be 1 mL, and excessive injection should be avoided to prevent balloon rupture.

9) Angiography should be performed after the hepatic vein is blocked by balloon and WHVP measurement is completed. In case of contrast agent reflux (indicating poor blocking), the balloon filling state should be adjusted, then re-angiography was performed; In case of veno-venous collateral shunts, the position of the balloon should be adjusted, or another hepatic vein should be selected for blocking.

10) Stable hemodynamics is crucial for pressure measurement, thus, the operator should patiently wait for a stable pressure reading before the next pressure measurement.

**Figure 2 Flow Chart**


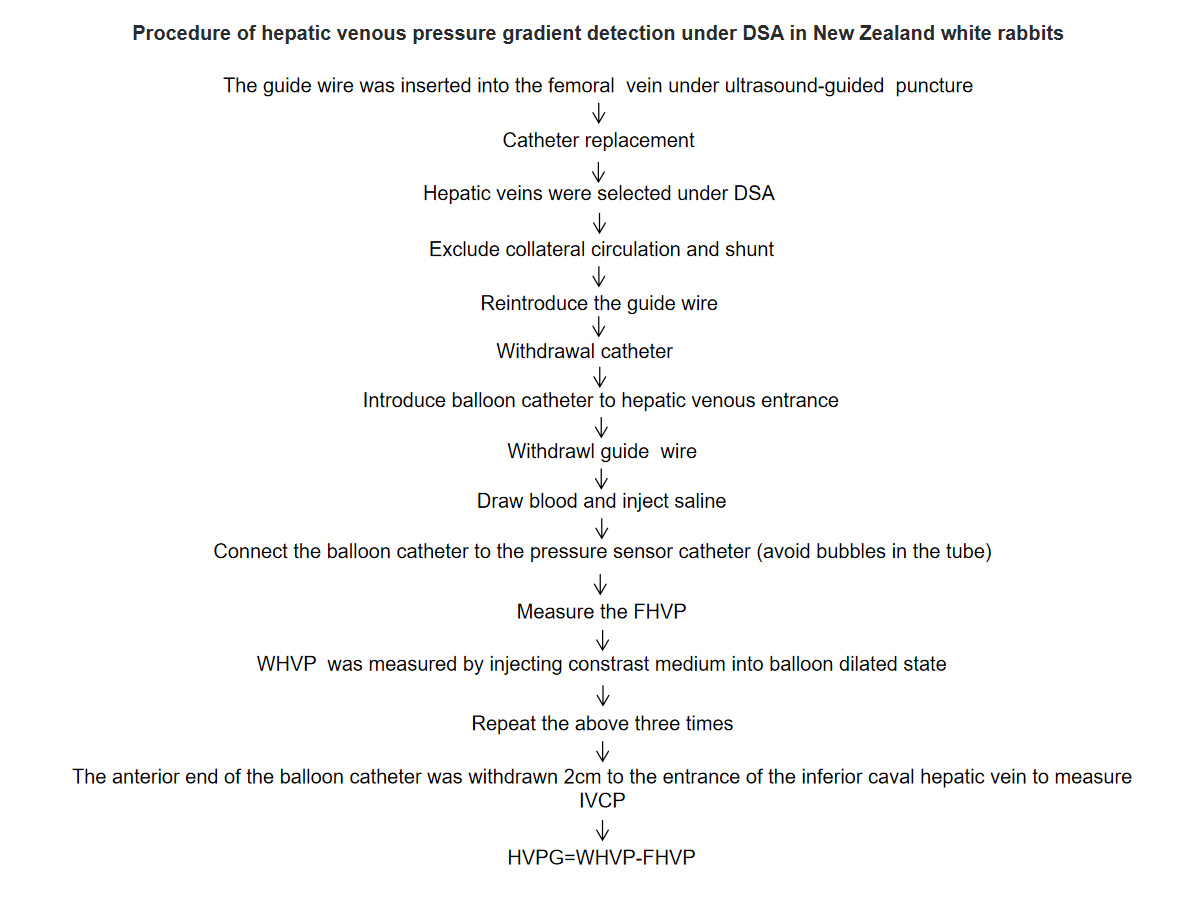

Supplement: S1 Appendix — (DOCX) [file pone.0305725.s001.docx]
